# A Comparative Study of Silicon Carbide Merged PiN Schottky Diodes with Electrical-Thermal Coupled Considerations

**DOI:** 10.3390/ma13112669

**Published:** 2020-06-11

**Authors:** Jiupeng Wu, Na Ren, Qing Guo, Kuang Sheng

**Affiliations:** College of Electrical Engineering, Zhejiang University, Hangzhou 310027, China; wujiupeng@zju.edu.cn (J.W.); guoqing@zju.edu.cn (Q.G.); shengk@zju.edu.cn (K.S.)

**Keywords:** SiC, MPS diode, junction temperature, surge current, thermal resistance

## Abstract

A comparative study of surge current reliability of 1200 V/5 A 4H-SiC (silicon carbide) MPS (Merged PiN Schottky) diodes with different technologies is presented. The influences of device designs in terms of electrical and thermal aspects on the forward conduction performance and surge current capability were studied. Device forward characteristics were simulated and measured. Standard single-pulse surge current tests and thermal impedance measurements were carried to show their surge capability and thermal design differences. An advanced thermal RC (thermal resistance-capacitance) model, with the consideration of current distribution non-uniformity effects, is proposed to accurately calculate the device junction temperature during surge events. It was found that a thinner substrate and a hexagonal layout design are beneficial to the improvement of the bipolar conduction performance in high current mode, as well as the surge current capability. The thinner substrate design also has advantages on thermal aspects, as it presents the lowest thermal resistance. The calculated failure temperature during the surge tests is consistent with the aluminum melting phenomenon, which is regarded as the failure mechanism. It was demonstrated that, for a SiC MPS diode, higher bipolar conduction performance is conducive to restraining the joule heat, and a lower thermal resistance design is able to accelerate the heat dissipation and limit the junction temperature during surge events. In this way, the MPS diode using a thinner substrate and advanced layout design technology is able to achieve 60% higher surge current density capability compared to the other technologies.

## 1. Introduction

The reliability of silicon carbide (SiC) devices deserves serious attention from device designers on account of the fact that SiC devices are generally designed to operate at high voltage/current conditions in harsh environments [1,2,3,4,5,6,7]. Surge current capability, which represents the ruggedness of power devices under high current pulses, is one of the key indices of the device reliability [8,9,10,11,12], for the reason that high current pulses are common at the starting-up of electrical equipment or during accidental circuit failures. Being commercially available since 2005 [8,13], SiC MPS diodes combine the advantages of a low forward voltage drop at nominal current and a high surge current capability. They gradually became the most promising type of SiC diodes in power applications [14,15]. The physical mechanism of the surge phenomenon and the device design methodology to improve the surge capability remain as one of the focused topics of research about SiC MPS diodes [16,17,18,19,20,21,22].

During the surge process, the flowing-in current pulse generates a high joule heat in the device, and thus the junction temperature rises. In most cases, the aluminum pad melting due to the high junction temperature is seen as the device failure mechanism [23,24,25,26]. Therefore, the main point to improve the surge capability of the MPS diodes is to limit the junction temperature rising. This could be achieved by optimizing the structure and layout designs to lower the forward voltage drop during the surge process, and hence to reduce the generated joule heat, which is an electrical aspect method. Many efforts have been focused on this aspect by simulations and experiments, and the influences of the layout patterns and parameters on the electrical characteristics at nominal and high currents are discussed [16,17,18,19,20,21,22]. Another way to limit the junction temperature is to ameliorate the thermal design of the device to accelerate the joule heat dissipation, which is a thermal aspect method. However, a systematic comparison considering the structure, layout and thermal designs among commercial SiC MPS diodes with different typical layout patterns has not been seen.

Moreover, since the junction temperature is a critical variable in the surge process, a good knowledge of it is necessary to investigate device surge capabilities. Unfortunately, the junction temperature is not a directly measurable physical quantity, and usually indirect measurement and simulation should be used [10,27,28,29]. Considering that the surge current phenomenon is a complicated electro-thermal coupled process, such simulations often tend to be extremely time- consuming. A physical model with electrical and thermal mechanisms considered would be more convenient. Although some electro-thermal models of the SiC MPS diodes have been established, they are usually behavioral macro-models and lack of physical insights [30,31,32]. Some authors use the compact RC model for the calculation of the junction temperature [33,34]. However, the current distribution inside the device has not been considered. According to the information the authors have, there is as yet no directly computable physical model for the determination of the junction temperature of SiC MPS diodes during the surge process.

In order to quantitatively investigate the influence of the structure, layout and thermal designs on the surge capability of SiC MPS diodes, a detailed comparative study was performed on three 1200 V/5 A commercial devices (denoted as Device 1, Device 2, and Device 3, respectively) from three manufacturers. The advantages of the hexagonal layout design are explained well, with physical insights. A simple, efficient and accurate model for the calculation of the junction temperature during the surge process was also developed. This paper is arranged as follows. In Section 2, measurement results of device structural parameters are listed. The device structures and physical models used for the device simulation are shown. The experimental setup for the forward characteristics and surge current tests are also presented. In Section 3, simulated and measured static forward characteristics are compared among the three devices. The correlations between the forward conduction performance and the device structure designs are analyzed in detail. Besides this, the surge current capabilities of the three devices are also compared and discussed. In Section 4, in order to demonstrate the influences of device designs in both electrical and thermal aspects on the surge current capability, an advanced thermal RC model is presented for the junction temperature calculation. The current distribution non-uniformity during the surge process is considered. The thermal resistance and capacitance of each material layer used in this model are obtained by thermal impedance measurement. Before being imported into the model, such measured thermal data are modified according to the current distribution in the device cell during the surge process. Analysis and device comparison are conducted based on the junction temperature calculation results. Finally, conclusions are presented in Section 5. It is demonstrated that a combination of good electrical and thermal design is able to restrain the rising of the junction temperature and to increase the surge current capability of MPS diodes significantly.

The symbols of variables used in this paper are explained in Table 1.

## 2. Materials and Methods

In this section, the decap measurement results of the structural parameters of the three devices (Device 1–3), such as the epitaxial layer and cell/layout design parameters, are clearly listed. The device structures of the three devices used for the forward characteristic simulation by the TCAD (Technology Computer Aided Design) software, as well as the applied physical models, are shown. The experimental setup for the forward characteristics and surge current tests are also presented.

### 2.1. Device Structural Parameters

**Figure 1:**
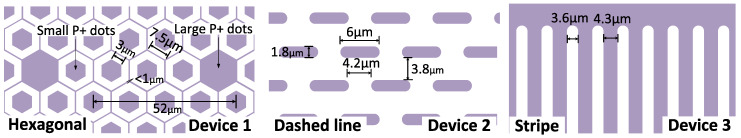
Layout designs of the three MPS diodes under test, drawn according to the microscopic observations. Colored regions stand for P+ regions in the active region. Device 1 has large P+ dots in its active region.

The chip sizes and layout schematic parameters were measured by scanning electron microscope (provided by Hitachi, Tokyo, Japan) after the decap of the three devices, as shown in Figure 1 and Table 2. The layout designs are quite typical for MPS diodes. The method used to estimate the epitaxy layer thickness (*t_epi_*) and doping concentration (*N_epi_*) is explained in Appendix A. It was noticed that Device 1 has the thinnest substrate and the smallest chip size. It also has additional large P+ regions placed periodically in the active region, which is reported to be able to improve the device’s high current conduction performance [27,35].

### 2.2. Device Simulation Structure and Models

**Figure 2:**
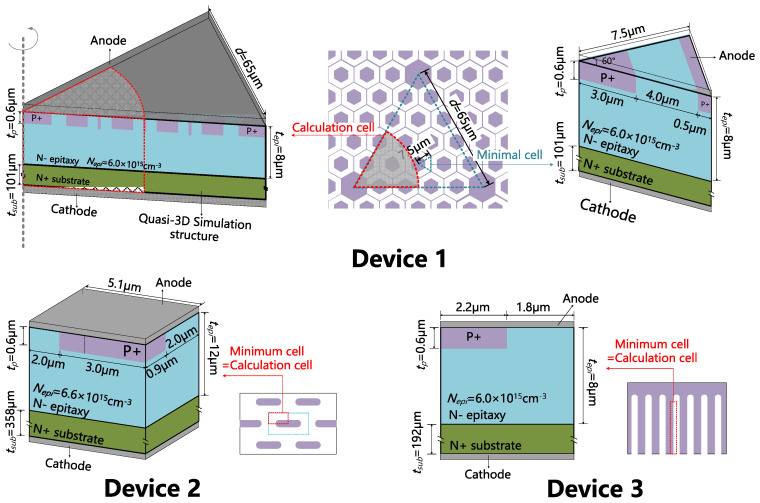
The simulation structures of the three devices. We define a ‘calculation cell’ and a ‘minimal cell’ for Device 1. The minimal cell is equivalent to the calculation cell for Device 2 and 3.

The cell structures of the three devices used for the forward characteristics simulation are shown in Figure 2, drawn according to the parameters extracted above. For Device 1, the minimal cell is a 1/6 hexagonal prism with a side length of 7.5 μm. Considering the complication that some of the minimal cells are replaced by large P+ dots, we define a ‘calculation cell’ as a 60°-cylinder to approximate the real layout design. For Device 2 or 3, the minimal cell is equivalent to its calculation cell. Simulations were performed on Silvaco^TM^ version 2018 (provided by Silvaco, Inc, Santa Clara, CA, USA), and the physical models used are listed in Table 3. For Device 1 or 2, 3D simulation is necessary, and for Device 3, 2D simulation is sufficient.

### 2.3. Experimental Setups of the Forward Characteristic Measurements and Serge Tests

**Figure 3:**
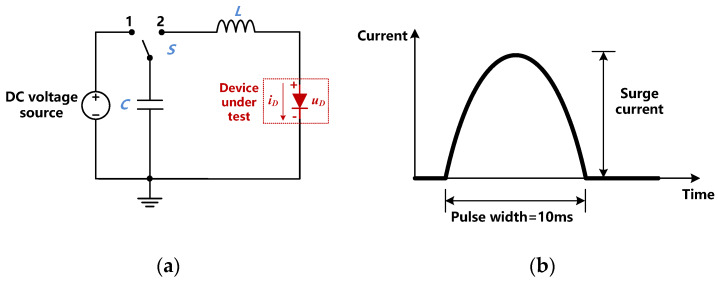
(**a**) The circuit of the test bench and (**b**) the waveform of the current pulse for the surge current capability tests.

The forward characteristics of the three devices at room and high temperatures were all measured by the curve tracer Tektronix B371 (provided by Tektronix, Inc, Beaverton, OR, USA) with a 250 μs-long voltage pulse. The high temperature circumstance was obtained with an oven. Surge current capability tests of the three devices were performed with a 10 ms-long half-sinusoidal pulse current source on a customized test bench (Figure 3). The magnitude of the current pulse was gradually raised until the failure of each device, which could be identified by the distortion of the voltage waveform. The peak current value of the current pulse for each surge test is denoted as the surge current. 

## 3. Results

In this section, the simulated and measured static forward characteristics are compared among the three devices. The correlations between the forward conduction performance and the device structure designs are analyzed in detail. The advantage of the hexagon layout design is clearly explained. The surge current capabilities of the three devices are also compared and discussed.

### 3.1. Simulated and Measured Results of the Forward Characteristics

**Figure 4:**
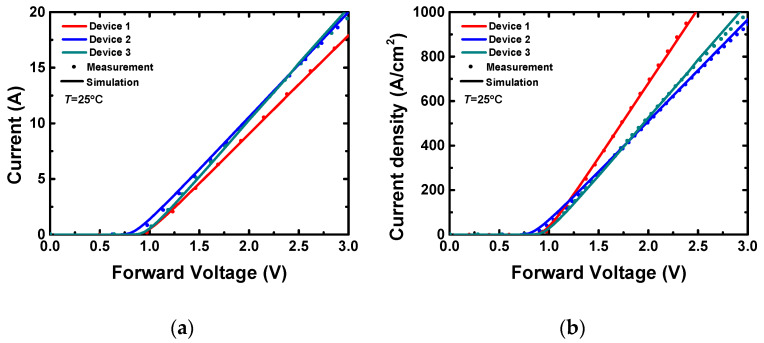
Simulated and measured forward characteristics of the three devices: (**a**) low current–voltage (I–V) curves, (**b**) low current density–voltage (J-V) curves.

The simulated and measured forward characteristics in the low current regime at room temperature are shown in Figure 4, as well as the measured ones. The simulated I–V (current–voltage) curves match well with the measured results (Figure 4a), indicating the credibility of the extracted device structural parameters. The current density calculated with the chip sizes and the J-V (current density–voltage) curves for the three devices is shown in Figure 4b. Among the three devices, Device 1 shows a higher current density at the same forward voltage, indicating that its thinner substrate helps to reduce the device’s on-specific resistance; thus, the same nominal current capability (5A in this study) can be achieved with a smaller chip area. On the other hand, Device 2 has the thickest epitaxy layer and the thickest substrate, and it outputs the lowest current density. It consumes the largest device area to achieve the 5A nominal current capability.

**Figure 5:**
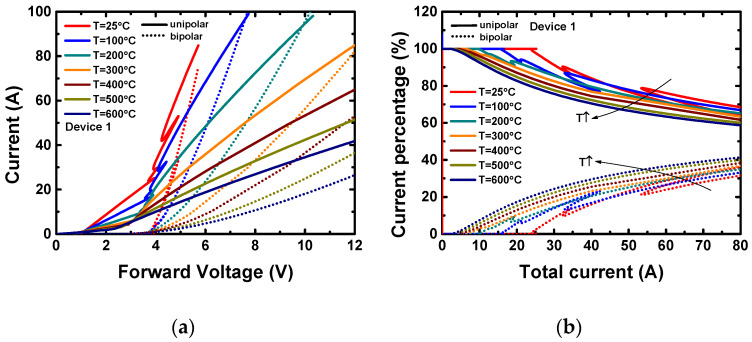
(**a**) The simulated static forward characteristics, with unipolar and bipolar current plotted separately, of Device 1 at *T* = 25–600 °C. (**b**) The percentage of unipolar and bipolar current in the total current of Device 1 extracted from (**a**).

The forward characteristics in the high current regime (up to 100 A) at room and high temperatures (up to 600 °C) were also simulated for the three devices. The unipolar and bipolar current components were separated by a dual-electrode setup in the simulation. The simulated I–V characteristics of Device 1 are shown in Figure 5a, in which the unipolar and bipolar current are plotted separately. The simulated I–V characteristics of all the three devices are shown in Figure S1a,c,e. The currents conducting through the electrodes above N- regions are defined as unipolar currents, and plotted as solid lines. The currents conducting through the electrodes above P+ regions are defined as bipolar currents, and plotted as dotted lines. The ripples on the unipolar I–V curves are caused by the injection of the minority carriers when the PN junction turns on. With the unipolar and bipolar current components, the percentages of the unipolar and bipolar current in total current can be calculated, and the results of Device 1 are also shown in Figure 5b. The percentages of the unipolar and bipolar current of all three devices are shown in Figure S1b,d,f. As the temperature rises, either the unipolar or bipolar current at the same forward voltage decreases. The decrease of the unipolar current comes from the fact that the electron mobility is a negative temperature coefficient [36]. On the other hand, the forward voltage corresponding to the bipolar current is mostly dominated by the substrate. Since the highly N-doped substrate is always unipolar conductive, the bipolar current follows the same rule on temperature as the unipolar current. Moreover, the higher the total current or the temperature, the higher the ratio the bipolar current takes. The reason is that the PN junction is more forward biased at a higher total current, and the intrinsic carrier density is increased with temperature. Both of the two factors increase the bipolar current under the same total current level. It is clear that the current distributes non-uniformly for all three devices. The unipolar current dominates the total current, whether the PN junction is activated or not. Due to its lowest P+ ratio (18%), Device 2 has the lowest bipolar conduction capability, and the bipolar current takes only 12% at 80 A at 600 °C. Contrarily, thanks to the hexagon layout design and the additional large P+ region design, Device 1 has the highest bipolar conduction capability. Its bipolar current takes 40% at 80 A at 600 °C, although its P+ ratio is not the highest.

**Figure 6:**
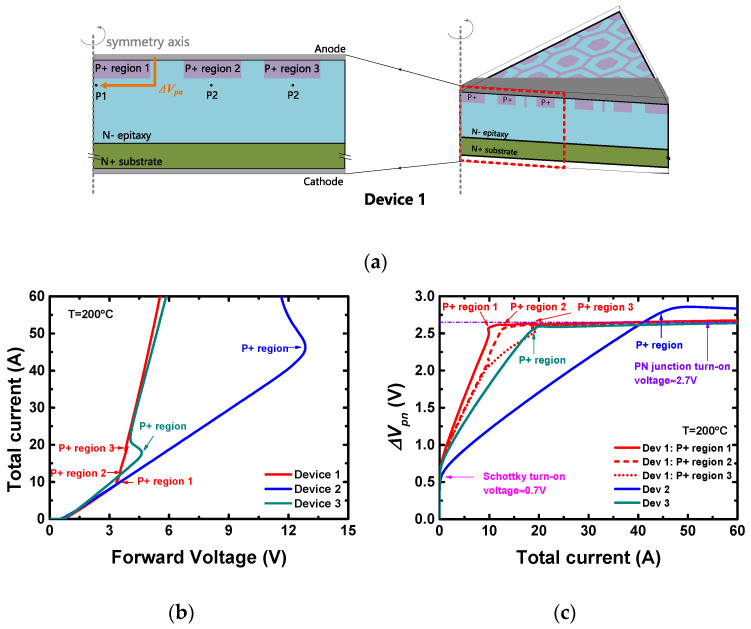
(**a**) The schematic diagram of the method of calculating the voltage drop of a PN junction in a cell of the MPS diode, showing Device 1 as an example. (**b**) The forward characteristics and (**c**) the dependence of the PN junction voltage drop on the total current (Δ*V_pn_-I* curves) of the three devices at *T* = 200 °C. The points corresponding to the activation of the PN junctions of each device are marked.

When compared with the other two layout designs (dashed line design and stripe design), the advantage of the hexagon layout design could be explained as follows. The PN junction in the MPS diodes is activated by the voltage drop generated by the current flowing along the path near the P+ region [27]. As shown in Figure 6a, the three P+ regions in the calculation cell of Device 1 are named as P+ region 1, 2 and 3, respectively. For each P+ region, a potential calculation point is placed below and near the junction (i.e., point P1, P2 and P3). The PN junction turns on when the voltage drop between the anode and the potential calculation point (e.g., Δ*V_pn_* for P+ region 1) is larger than its turn-on voltage. Since the potential calculation points are near the PN junctions, Δ*V_pn_* is affected only by the current and the layout design, independent of the epitaxial and substrate layers. The same rule also goes for the P+ regions in Devices 2 and 3.

According to the method discussed above, the simulated forward characteristics and the dependence of the PN junction voltage drop on the total current (Δ*V_pn_*-*I* curves) of the three devices at *T* = 200 °C are plotted in Figure 6b,c, respectively. The points corresponding to the activation of the PN junctions of each device are marked. The voltage drop rises linearly with the total current before the activation of the PN junction, until it reaches the PN junction turn-on voltage (~2.7 V at *T* = 200 °C) and stays constant. The slope of the Δ*V_pn_*-*I* curve represents the efficiency of the total current to bias a PN junction. Figure 6c shows that Device 1 has the highest efficiency, in which P+ region 1 is biased the most efficiently and is the earliest to turn on. This could be explained by the fact that P+ region 1 in Device 1 has the largest lateral dimension (6.5 μm), and the same current generates a higher voltage drop along a longer path. Moreover, for the same lateral dimension, the P+ region with a hexagon design occupies a much smaller area than the dash line design and stripe design. Thus, the hexagon layout design is superior to the other two designs.

**Figure 7:**
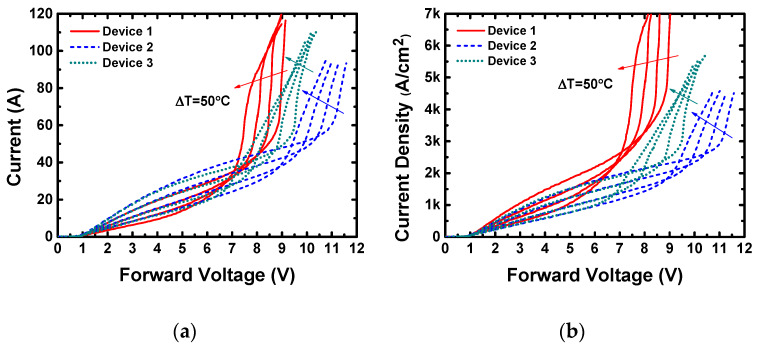
(**a**) The measured forward I–V characteristics of the three devices at *T* = 25–175 °C with a step of Δ*T* = 50 °C. (**b**) Current density–voltage (J-V) characteristics calculated from (**a**).

**Figure 8:**
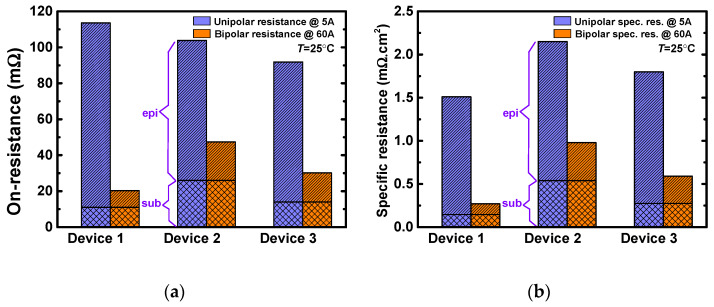
(**a**) The on-resistance (*R_on_*) and (**b**) the specific on-resistance (*R_on,sp_*) of the three devices in unipolar mode (5 A) and bipolar mode (60 A) at *T* = 25 °C, extracted from Figure 3, with the resistance of the epitaxy layer and substrate shown separately.

After the simulation study, the high current forward I–V characteristics were measured at different temperatures (*T* = 25–175 °C, Δ*T* = 50 °C), and the results are presented in Figure 7a. In addition, Figure 7b shows the current density–voltage (J-V) characteristics calculated from Figure 7a. The I–V curves are not exactly the same as the simulated ones shown in Figure 5, since the simulation is an isothermal process, while the self-heat is inevitable in measurement, and the minority carrier lifetime is much shorter than that in the simulation. However, the qualitative difference of the forward characteristics is the same. Device 1 has the highest bipolar conductive capability, while Device 2 has the lowest. This fact shows distinctly the advantage of the thinner substrate and the hexagon layout design. The on-resistances (*R_on_*) and specific on-resistances (*R_on,sp_*) of the unipolar (5 A) and bipolar (60 A) modes at room temperature extracted from Figure 7 are summarized in Figure 8. Although the *R_on_* of Device 1 is slightly higher than the other two devices in unipolar mode, it decreases to the lowest in bipolar mode, showing an excellent high current conduction capability. As for *R_on,sp_*, the *R_on,sp_* of Device 1 is the lowest, thanks to its smallest substrate thickness. At 60 A, the *R_epi,sp_* of Device 1 is the lowest, and thus its *R_on,sp_* is also the smallest, indicating a good conduction capability at high current.

### 3.2. Surge Current Capabilities

**Figure 9:**
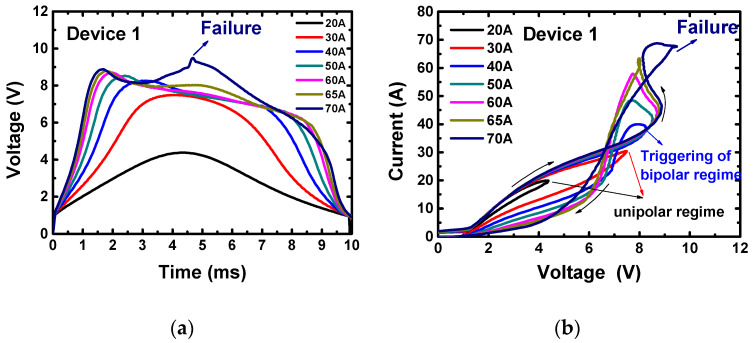
(**a**) Voltage waveforms and (**b**) I–V trajectories of Device 1 during the surge current tests.

**Figure 10:**
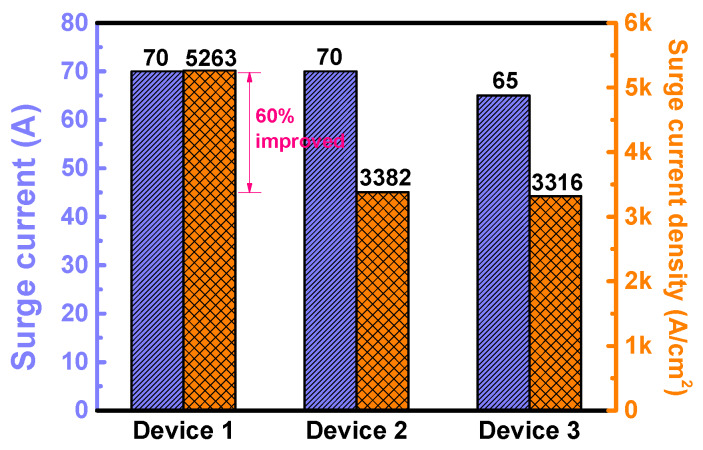
The surge current capability (left axis) and surge current density capability (right axis) of the three devices.

The voltage waveforms and I–V trajectories of Device 1 during the surge current tests are shown in Figure 9a,b, respectively. The data of all the three devices are shown in Figure S2. The three diodes have quite similar surge behaviors. When the current is less than 30 A, the devices work in unipolar mode. When the current is larger than 40 A, they enter the bipolar mode, with a negative resistance branch on the I–V trajectory. Devices 1 and 2 have the same surge capability (70 A), while Device 3 has a slightly lower surge capability (65 A). Considering their chip area differences, the surge current density capability of Device 1 is ~60% higher than the other two devices, as summarized in Figure 10.

## 4. Discussion

In this section, to discuss the influences of device designs in both electrical and thermal aspects on the surge current capability of the MPS diodes, an advanced thermal RC model is presented for junction temperature calculation in surge conditions, with current distribution non-uniformity considered. The thermal resistance and capacitance of each material layer was extracted from thermal impedance measurement results, and then modified according to the current distribution non-uniformity explained in Section 3.1. After that, the thermal data, as well as the measurement results of the current and voltage waveforms, were imported into the RC model to calculate the junction temperature during the surge process. The calculation results are discussed and analyzed.

### 4.1. Thermal Impedance Measurement

In order to compare the thermal designs of the three devices, their thermal impedance was measured on a Mentor Graphics Power Tester 1500A (provided by Mentor Graphics Corporation, Wilsonville, OR, USA), according to the test standard JESD51-14 [38]. The junction-to-case thermal impedance was extracted from the structure function (Figure S3a) [38,39], which was calculated from the cooling curves. It was observed that the thermal impedance of Devices 2 and 3 is quite similar, and they have close junction-to-case thermal resistances (1.43 K/W and 1.54 K/W, respectively). On the other hand, the structure function curve of Device 1 deviates from those of Devices 2 and 3, and it has the lowest junction-to-case thermal resistances (1.04 K/W).

**Figure 11:**
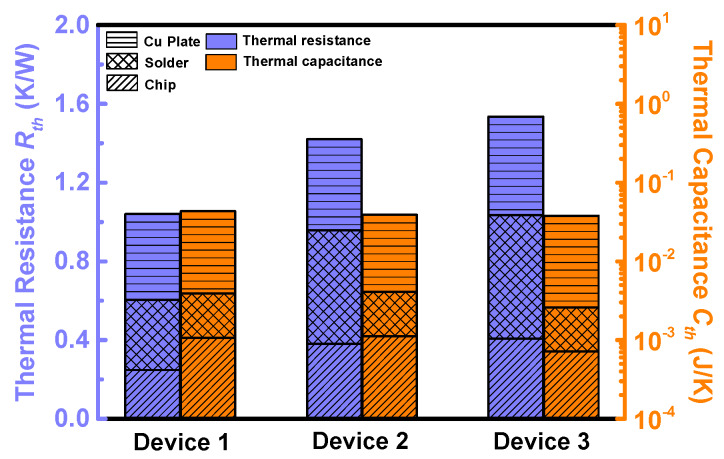
The thermal resistance (left axis) and capacitance (right axis) of each material layer (chip, solder and Cu plate).

In order to compare the thermal designs and package technology of the three devices, the thermal resistance (*R_th_*) and capacitance (*C_th_*) of each material layer (i.e., the chip, the solder and the copper plate) of the three devices were extracted from the structure functions [39,40], as shown in Figure S3b–d. The extracted results are summarized in Figure 11. Device 1 has a lower *R_th_* in both chip and solder layers. It is the lowest *R_th_* of the chip layer and the solder layer that largely decrease the total *R_th_* of Device 1. The *R_th_* of the copper plate and *C_th_* of all the three layers are quite similar among the three devices. 

### 4.2. Thermal RC Model for Junction Temperature Calculation

**Figure 12:**
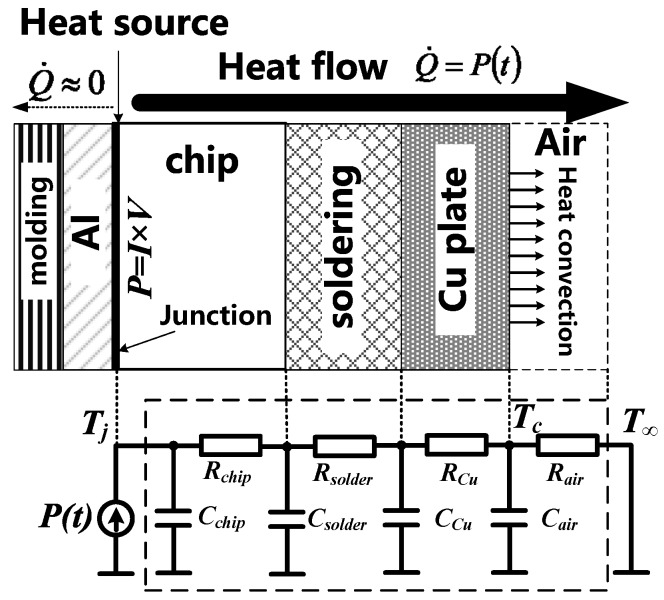
The compact thermal RC model of a packaged device. Each RC component stands for a layer of material. The power source stands for the heating power at the junction.

In order to calculate the junction temperature during the surge tests, a compact thermal RC model from the junction to the ambient was established [41], as illustrated in Figure 12. Each layer was abstracted as a thermal capacitor and a thermal resistor. The thermal circuit transfer function was calculated from the thermal capacitor/resistor values. The input heating power was compact at the junction, and its power value was calculated as the measured current multiplied by the device voltage. The junction temperature was acquired by the convolution between the heating power and the transfer function.

**Figure 13:**
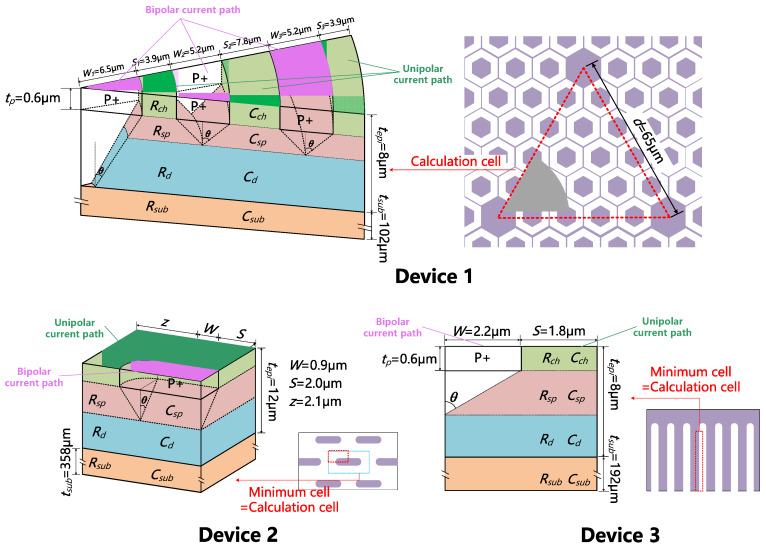
The current paths in a calculation cell of the three devices, which is divided into four parts (channel, spreading, drift and substrate), as labeled with different colors.

For the traditional method, the extracted thermal resistance and capacitance are directly imported into this model to calculate the transfer function. However, this method tends to underestimate the junction temperature. As in the aforementioned thermal impedance measurement method in Section 4.1, the thermal parameters were calculated from the cooling curve as the device cools down from a steady thermal state. Such a fact indicates that the whole chip acts as the heat flow path during the thermal impedance measurement, as shown in Figure S4a. However, the surge process is a transient thermal process, and it is reasonable to deduce that only part of the chip area conducts the current, as shown in Figure S4b. From the simulated static forward characteristics shown in Figure 5, since the unipolar current dominates the total current within the current range of 0–80 A, it can be assumed that the current during the surge process flows into the device mostly through the N- region, and then spreads and flows out of the device through the cathode, as the colored regions illustrate in Figure 13.

(1) $$\kappa =\frac{t_{epi}+t_{sub}}{A_{chip}R_{chip}}$$

(2) $$C_{V}=\frac{C_{chip}}{A_{chip}\left(t_{epi}+t_{sub}\right)}$$

(3) $$R_{tot}=\left(R_{ch}+R_{sp}+R_{d}+R_{sub}\right)/m$$

(4) $$C_{tot}=\left(C_{ch}+C_{sp}+C_{d}+C_{sub}\right)m$$

**Figure 14:**
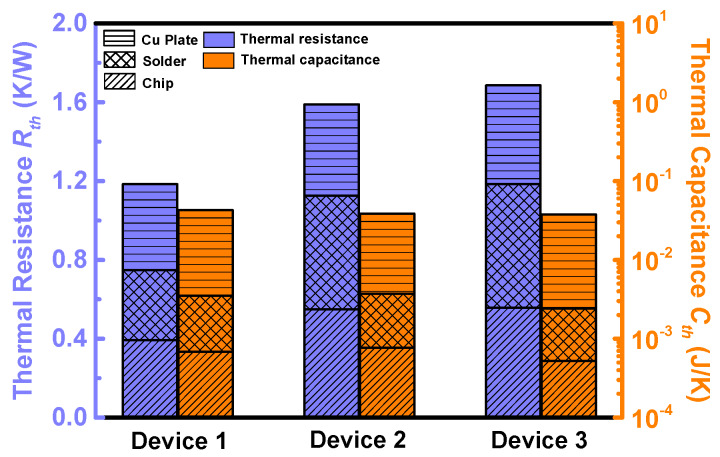
The modified thermal resistance and capacitance of each material layer of the three devices.

The thermal resistance and capacitance extracted in Figure 11 should be modified according to the current path shown in Figure 13, before being imported into the RC model to calculate the transfer function. The current path shown can be divided into four parts (the channel part, the spreading part, the drift part and the substrate part). The thermal resistance and capacitance of each part in one calculation cell of the three devices can be calculated by the equations shown in the Appendix B. The thermal conductivity and the specific volumetric heat capacity of the chip can be calculated by Equation (1) and (2); the total thermal resistance and capacitance of the chip can be calculated by Equation (3) and (4). The modified thermal impedance data are shown in Figure 14. Compared with the results in Figure 11, the thermal resistance of the chip has risen by 58%, 47% and 36%, respectively; the thermal capacitance has decreased by 36%, 31%, and 27%, respectively. Although the current non-uniformity increases the chip’s thermal resistance, the total thermal resistance of Device 1 is still the lowest.

**Figure 15:**
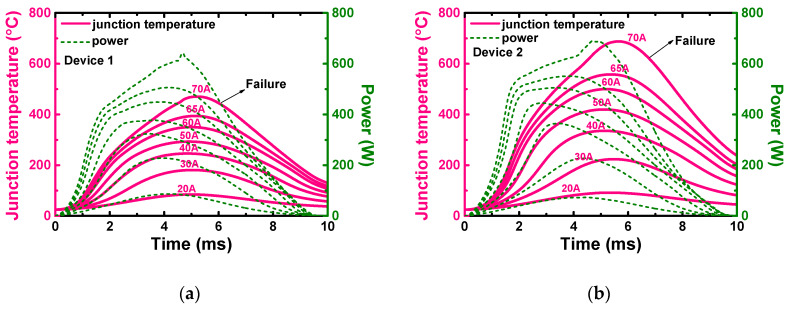

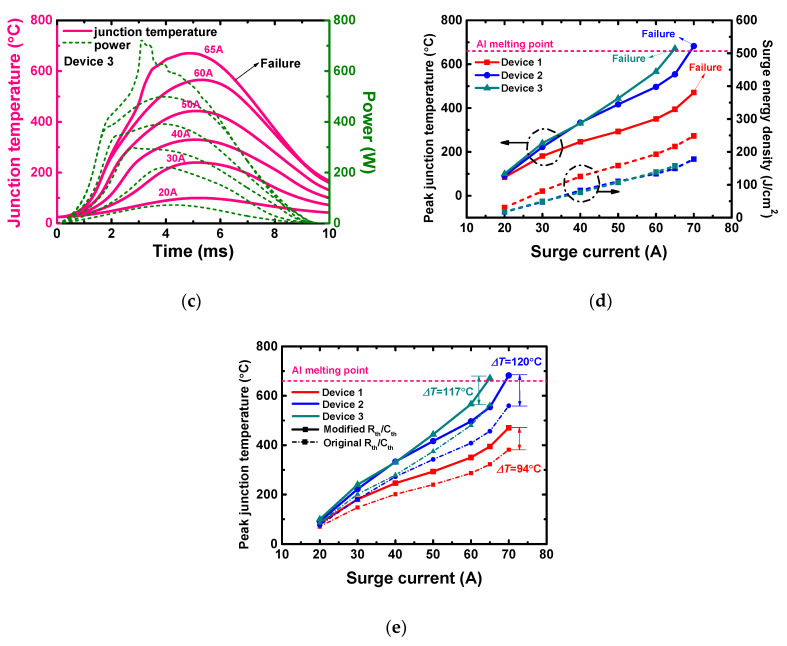
(**a**–**c**) The junction temperature and surge heating power of the three devices during the surge tests in Section 2.3, calculated by importing the thermal parameters in Figure 14 into the compact RC model. (**d**) The peak junction temperature and the surge energy density during the surge tests of the three devices. (**e**) The comparison of the peak junction temperature, calculated by the modified thermal impedance in Figure 14 and by the original thermal impedance in Figure 11 of the three devices.

Based on the presented thermal RC model, which considers the current distribution non-uniformity effects, the calculated junction temperatures during the surge process of the three devices are shown in Figure 15a–c, as well as the surge heating power of each surge test. The waveforms of the heating power and the junction temperature are all unimodal pulses, while the peak junction temperature (*T_j,max_*) appears at *t* = 5–6 ms, which is 1–2.5 ms later than the heating power peak. The *T_j,max_* and the surge energy density at different surge currents are summarized in Figure 15d. Owing to its excellent thermal design, the *T_j,max_* of Device 1 is lower than Devices 2 and 3 at any surge current, despite the fact that it dissipates the highest joule heat per unit area. The failure of Devices 2 and 3 happened when the junction temperature reached the aluminum melting point (660 °C), indicating that the melting of the anode electrode is the main reason for the devices’ failure [23,24,25,26]. As a comparison between the traditional and the advanced thermal RC models, the peak junction temperatures of the three devices calculated by the original thermal impedance (shown in Figure 11) are also plotted together with those demonstrated in Figure 15d (i.e., calculated by the modified thermal impedance shown in Figure 14), as shown in Figure 15e. It is obvious that the peak junction temperature calculated by the original thermal impedance is greatly underestimated, by about 100 °C for the three devices. None of the three devices experienced a peak junction temperature higher than the aluminum melting point at the last surge test, which is contrary to the fact that the device failed in this test. Thus, the necessity of the application of the advanced thermal RC model was verified.

**Figure 16:**
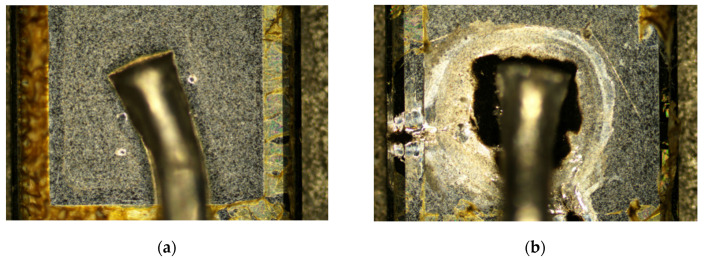
The decap microscopic photos of the devices without and with surge current tests. (**a**) The device is fresh, without any test or measurement, and the anode metal remains intact. (**b**) The device failed during the surge test, and the melting of the anode metal could be observed.

The melting of the anode electrode was also confirmed by the device decap microscopic photos of surge untested and tested devices, as shown in Figure 16. Compared with the untested device (Figure 16a), the anode metal of the surge-destroyed device (Figure 16b) showed a clear circular melting imprint around the boding wire. As for Devices 2 and 3, the advanced thermal RC model gave accurate junction temperature results consistent with the experimental facts. However, the peak junction temperature of Device 1 at failure was much lower than 660 °C, implying that some secondary effects should be considered, which is beyond the capability of the one-dimensional compact RC model.

### 4.3. Analysis

Device 1 achieved a higher bipolar conduction capability than the other two devices by possessing the thinnest substrate and the hexagon layout design with large P+ dots. Although the simulation results in Figure 5 show that, for all the devices, only a small part of the total current is conducted by the P+ regions, the large P+ dots in Device 1 turn on at a relatively lower current, and undertake a higher percentage current than the other two devices. It also has the lowest junction-to-case thermal resistance, even if the high current distribution non-uniformity is considered. Thus, the surge current generating heat and the junction temperature could both be kept low, and a higher surge current capability could be achieved. The device failure mechanism during the surge process was verified by the junction temperature calculation results of Devices 2 and 3, while those of Device 1 deviated somehow from our expectation. A one-dimensional compact RC model with constant thermal resistance and capacitance may not be qualified enough to handle the situation of Device 1, in which the complicated hexagon cell may introduce some secondary effect, which deserves future research.

## 5. Conclusions

A comparative study of three commercial 1200 V/5 A MPS diodes from both electrical and thermal considerations was conducted in this paper. The structural parameters and layout designs of the three devices were measured and extracted. The simulated low current forward characteristics based on the design parameters were consistent with the measured ones, indicating their credibility and laying the foundation of the following analysis. The measured high current forward characteristics demonstrated the high current conduction advantage of Device 1, which had a hexagonal layout design and a thinner substrate. The bipolar on-resistance and specific on-resistance of Device 1 stay the lowest among the three devices, which means that the surge current generating heat could be minimized. This accords with the electrical aspects in order to increase the surge current capability mentioned in the introduction. The surge current capability of the three devices was tested under a 10 ms-long half-sinusoidal current pulse. The three devices have quite similar surge capability, while Device 1 has a ~60% higher surge capability density than the other two devices, indicating that Device 1 has achieved the same surge capability with a smaller chip size. The thermal impedance of the three devices was measured, and the thermal resistance and capacitance of each material layer of the package were extracted. While the three devices have basically the same thermal capacitance, Device 1 has the lowest thermal resistance due to its lower chip and solder thermal resistance. The current non-uniformity effects during the surge tests were considered, and the thermal resistance/capacitance was modified accordingly for each device. The modified thermal resistance of Device 1 is the lowest. Thus, even though Device 1 dissipates the highest surge energy density during the surge tests due to its smallest chip size, its junction temperature stays lowest at the same surge current when compared to the other two devices, indicating its excellent thermal design. This accords with the aforementioned thermal aspects to increase the surge current capability. The melting of the aluminum layer was observed by microscope after device decap, which verified the surge failure mechanism i.e., anode metal melting. The failure junction temperature was calculated to be 660 °C, which is close to the aluminum melting point. Thus, the accuracy of the proposed thermal RC model, with consideration of current distribution non-uniformity, was verified. In a word, a combination of good electrical and thermal design is able to increase the surge current capability of MPS diodes significantly.

## Figures and Tables

**Table 1:** Symbols of variables used in this paper.

Variable	Definition	Variable	Definition
*N_epi_*	Doping concentration of the epitaxy layer	*C_chip_*	Steady thermal capacitance of the chip
*t_p_*	Depth of the P+ region	*R_ch_*	Channel thermal resistance
*t_epi_*	Thickness of the epitaxy layer	*C_ch_*	Channel thermal capacitance
*t_sub_*	Thickness of the substrate	*R_sp_*	Spreading thermal resistance
*A_act_*	Area of the active region	*C_sp_*	Spreading thermal capacitance
*A_chip_*	Area of the chip	*R_d_*	Drift thermal resistance
*A_cell_*	Area of the calculation cell	*C_d_*	Drift thermal capacitance
*m*	Number of the calculation cell, *m* = *A_act_*/*A_cell_*	*R_sub_*	Substrate thermal resistance
*κ*	Thermal conductivity of the chip	*C_sub_*	Substrate thermal capacitance
*C_V_*	Specific volumetric heat capacity	*R_tot_*	Total thermal resistance of the device for junction temperature calculation
*θ*	Current path spreading angle	*C_tot_*	Total thermal capacitance of the device for junction temperature calculation
*R_chip_*	Steady thermal resistance of the chip	*T*	Temperature

**Table 2:** Device structural parameters and layout designs.

Cross-Section View of Chips	Dev	*t_epi_* (μm)	*N_epi_* (cm^−3^)	*t_sub_* (μm)	*A_act_* (mm^2^)	Layout Design	P+ Ratio
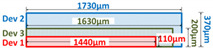	1	8.0	6.0 × 10^15^	102	1.33	Hexagon	35%
	2	12.0	6.6 × 10^15^	358	2.07	Dash line	18%
	3	8.0	6.0 × 10^15^	192	1.96	Stripe	54%

**Table 3:** Physical models used for device forward characteristic simulation [36,37].

Model Classification	Models Used in Simulation	Description
Carrier statistics models	fermi-dirac	Energy state occupation probability model with fermi-dirac statics
	bgn (Bandgap narrowing)	Doping-dependent bandgap width model
Carrier mobility model	analytical	Doping- and temperature- dependent mobility model
	fldmob	Electrical-field-dependent mobility model
Recombination model	Shockley-Read-Hall (SRH)	Indirect recombination mechanism with two carriers and a recombination center involved. Important for SiC.
	Auger	Direct recombination mechanism with three carriers involved. Important at high current densities.

**Figure A1:**
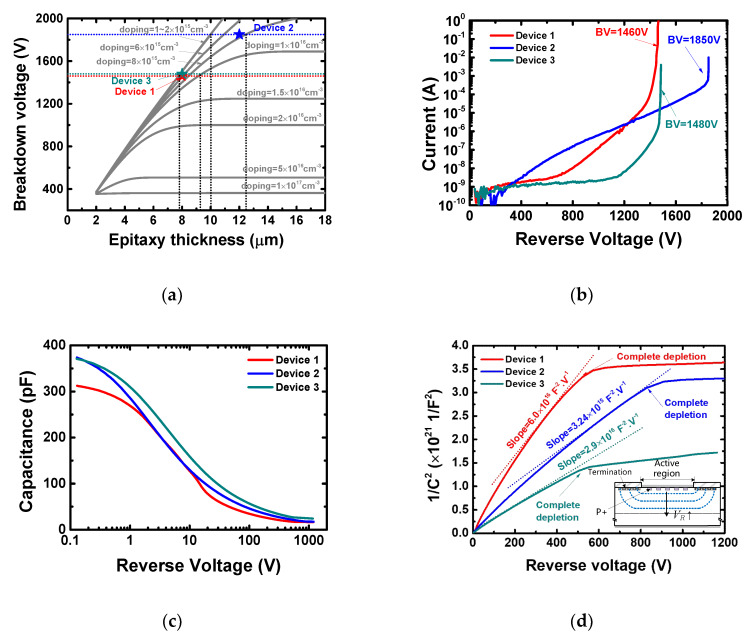
(**a**) The dependence of the breakdown voltage (*BV*) on the epitaxy layer thickness (*t_epi_*) and doping concentration (*N_epi_*) by simulation. (**b**) The measured reverse I–V characteristics, (**c**) the measured capacitance–voltage (C-V) characteristics, and (**d**) the 1/C^2^-V curves of the three devices. The inset in (**d**) shows the extension of the depletion region as the reverse bias voltage (*V_R_*) increases.

## Supplementary Materials

The following are available online at https://www.mdpi.com/1996-1944/13/11/2669/s1, Figure S1: The simulated static forward characteristics, with unipolar and bipolar current plotted separately, of (**a**) Device 1, (**c**) Device 2, and (**e**) Device 3 at *T* = 25–600 °C. The percentage of unipolar and bipolar current in total current of (**b**) Device 1, (**d**) Device 2, and (**f**) Device 3, extracted from (**a**), (**c**) and (**e**), Figure S2: Voltage waveforms and I-V trajectories of the three devices during the surge current tests: (**a**,**b**) for Device 1, (**c**,**d**) for Device 2, and (**e**,**f**) for Device 3, Figure S3: (**a**) The structure functions of the three devices. The junction-to-case thermal impedance could be determined by the bifurcation point of the solid and dashed lines. The zoomed-in structure function and its derivative of (**b**) Device 1, (**c**) Device 2, and (**d**) Device 3, as well as the extracted thermal resistance and capacitance of each layer, Figure S4: The heat paths inside the chip, the solder, and the copper plate of the device during (**a**) the thermal impedance measurement and (**b**) the surge process.

## Appendix A

The epitaxy layer thickness was estimated by its breakdown voltage. The dependence of the breakdown voltage (*BV*) on the epitaxy layer thickness (*t_epi_*) and doping concentration (*N_epi_*) was acquired by simulation, as shown in Figure A1a. The reverse I–V characteristics of the three devices were measured by the curve tracer Tektronix B371, as shown in Figure A1b. The avalanche *BV* of Device 1, 2, and 3 could be read out as 1460 V, 1480 V and 1850 V, respectively. According to Figure A1a, the *N_epi_* of Devices 1 or 3 should not be higher than 1 × 10^16^ cm^−3^, and that of Device 2 should not be higher than 8 × 10^15^ cm^−3^. Thus, the *t_epi_* range which satisfies these breakdown voltages can be estimated as 7.5–8.5 μm for Devices 1 or 3, and 10.0–12.5 μm for Device 2.

The doping concentration can be estimated by the capacitance–voltage (C-V) characteristics of the device. According to the theory of the abrupt P+N junction (almost the same as the Schottky junction), the reverse of the square of the capacitance (1/C^2^) and the reverse bias voltage (*V_R_*) satisfy

(A1) $$\frac{1}{C^{2}\left(V_{R}\right)}=\frac{2}{qA^{2}\varepsilon N_{epi}}\times V_{R}$$

where *A* is the area of the junction. Thus, 1/C^2^ depends linearly on *V_R_*. Donate the slope of the 1/C^2^-V curves as *η*, and *N_epi_* can be calculated as

(A2) $$N_{epi}=\frac{2}{qA^{2}\varepsilon \eta }$$

The C–V characteristics of the three devices were measured by the curve tracer Agilent B1505A, as shown in Figure A1c. The 1/C^2^-V curves are shown in Figure A1d. The slope of the 1/C^2^–V curve decreases to near zero when the epitaxy layer depletes completely. Since the P+ regions in the active region of the real device make the C–V and 1/C^2^–V curves deviate from those of the ideal parallel junction, it is recommended to use the part of the 1/C^2^-V curve which is near the complete depletion point. Considering that the depletion region extends under the termination region as *V_R_* increases during the C–V characteristic measurement (as shown by the inset in Figure A1d), the junction area *A* in Equation (A2) should be the device area instead of the active area. The device area of Devices 1, 2 and 3 are 2.07 mm^2^, 2.90 mm^2^ and 2.60 mm^2^, respectively. The calculated doping concentration of Devices 1, 2 and 3 are 5.7 × 10^15^ cm^−3^, 5.4 × 10 ^15^ cm^−3^ and 6.9 × 10^15^ cm^−3^, respectively. Considering all the secondary effects that make the C–V characteristics deviate from the ideal parallel junction, these calculated doping concentrations are only approximate values. By rechecking Figure A1a, it can be estimated that the doping concentrations/thicknesses of the three devices are (6–7) × 10^15^ cm^−3^/8 μm, (6–7) × 10^15^ cm^−3^/12 μm and (6–7) × 10^15^ cm^−3^/8 μm, respectively. More accurate values could be obtained by adjusting the *t_epi_* and *N_epi_* of the simulation structures shown in Figure 2 in the main text, until the simulated I–V curves match the measured ones, as shown in Figure 4 in the main text.

## Appendix B

The thermal resistance and capacitance of the channel part, the spreading part, the drift part and the substrate part in one calculation cell of the three devices can be calculated by the following equations, which are derived with the method proposed in [42,43]. Equations (A3)–(A5) are for Device 1, 2 and 3, respectively.

(A3) $$\{\begin{array}{l} A_{ch}=\pi \left[{\left(W_{1}+S_{1}\right)}^{2}-W_{1}{}^{2}\right]\\ +\pi \left[{\left(W_{1}+S_{1}+W_{2}+S_{2}\right)}^{2}-{\left(W_{1}+S_{1}+W_{2}\right)}^{2}\right]\\ +\pi \left[{\left(W_{1}+S_{1}+W_{2}+S_{2}+W_{3}+S_{3}\right)}^{2}-{\left(W_{1}+S_{1}+W_{2}+W_{2}+S_{2}+W_{3}\right)}^{2}\right]\\ A_{sp}\left(y\right)=\pi \left[{\left(W_{1}+S_{1}+y\tan\theta \right)}^{2}-{\left(W_{1}-y\tan\theta \right)}^{2}\right]\\ +\pi \left[{\left(W_{1}+S_{1}+W_{2}+S_{2}+y\tan\theta \right)}^{2}-{\left(W_{1}+S_{1}+W_{2}-y\tan\theta \right)}^{2}\right]\\ +\pi \left[{\left(W_{1}+S_{1}+W_{2}+S_{2}+W_{3}+S_{3}\right)}^{2}-{\left(W_{1}+S_{1}+W_{2}+W_{2}+S_{2}+W_{3}-y\tan\theta \right)}^{2}\right]\\ A_{d}\left(y\right)=\pi \left[{\left(W_{1}+S_{1}+W_{2}+S_{2}+W_{3}+S_{3}\right)}^{2}-{\left(W_{1}-y\tan\theta \right)}^{2}\right]\\ R_{ch}=\frac{t_{p}}{\kappa A_{ch}}\;C_{ch}=C_{V}t_{p}A_{ch}\\ R_{sp}=\frac{1}{\kappa }\int_{0}^{\frac{W_{2}}{2\tan\theta }}\frac{dy}{A_{sp}\left(y\right)}\;C_{sp}=C_{V}\int_{0}^{\frac{W_{2}}{2\tan\theta }}A_{sp}\left(y\right)dy\\ R_{d}=\frac{1}{\kappa }\int_{\frac{W_{2}}{2\tan\theta }}^{t_{epi}-t_{p}}\frac{dy}{A_{d}\left(y\right)}\;C_{d}=C_{V}\int_{\frac{W_{2}}{2\tan\theta }}^{t_{epi}-t_{p}}A_{d}\left(y\right)dy\\ R_{sub}=\frac{t_{sub}}{\kappa \pi {\left(W_{1}+S_{1}+W_{2}+S_{2}+W_{3}+S_{3}\right)}^{2}}\\ C_{sub}=C_{V}\pi t_{sub}{\left(W_{1}+S_{1}+W_{2}+S_{2}+W_{3}+S_{3}\right)}^{2} \end{array}$$

(A4) $$\{\begin{array}{l} A_{ch}=\left(W+S\right)\left(W+S+z\right)-\left(Wz+\frac{\pi W^{2}}{4}\right)\\ A_{sp}\left(y\right)=\left(W+S\right)\left(W+S+z\right)-\left(W-y\tan\theta \right)z-\frac{\pi {\left(W-y\tan\theta \right)}^{2}}{4}\\ R_{ch}=\frac{t_{p}}{\kappa A_{ch}}\;C_{ch}=C_{V}A_{ch}t_{p}\\ R_{sp}=\frac{1}{\kappa }\int_{0}^{\frac{W}{\tan\theta }}\frac{dy}{A_{sp}\left(y\right)}\;C_{sp}=C_{V}\int_{0}^{\frac{W}{\tan\theta }}A_{sp}\left(y\right)dy\\ R_{d}=\frac{t_{epi}-t_{p}-\frac{W}{\tan\theta }}{\kappa \left(W+S\right)\left(z+S+W\right)}\;C_{d}=C_{V}\left(t-t_{p}-\frac{W}{\tan\theta }\right)\left(W+S\right)\left(z+S+W\right)\\ R_{sub}=\frac{t_{sub}}{\kappa \left(W+S\right)\left(z+S+W\right)}\;C_{sub}=C_{V}\left(W+S\right)\left(z+S+W\right)t_{sub} \end{array}$$

(A5) $$\{\begin{array}{l} R_{ch}=\frac{t_{p}}{\kappa Sz}\;C_{ch}=C_{V}Szt_{p}\\ R_{sp}=\frac{1}{\kappa z}\int_{0}^{\frac{W}{\tan\theta }}\frac{dy}{\left(y\tan\theta +S\right)}\;C_{sp}=C_{V}z\int_{0}^{\frac{W}{\tan\theta }}\left(y\tan\theta +S\right)dy\\ R_{d}=\frac{t_{epi}-t_{p}-\frac{W}{\tan\theta }}{\kappa \left(W+S\right)z}\;C_{d}=C_{V}\left(t_{epi}-t_{p}-\frac{W}{\tan\theta }\right)\left(W+S\right)z\\ R_{sub}=\frac{t_{sub}}{\kappa \left(W+S\right)z}\;C_{sub}=C_{V}\left(W+S\right)zt_{sub} \end{array}$$
