# Supplementary material for: A Comparative Study of Silicon Carbide Merged PiN Schottky Diodes with Electrical-Thermal Coupled Considerations
Source: Materials (Basel). 2020 Jun 11;13(11):2669. doi: 10.3390/ma13112669 (PMC7321647; doi:10.3390/ma13112669)
Supplement: Supplementary file 1 [file materials-13-02669-s001.pdf]

materials

MDPI

Supporting information

A Comparative Study of Silicon Carbide Merged PiN Schottky Diodes with Electrical-Thermal Coupled Considerations

Jiupeng Wu, Na Ren *, Qing Guo and Kuang Sheng

College of Electrical Engineering, Zhejiang University, 310027 Hangzhou, China; wujiupeng@zju.edu.cn (J.W.); guoqing@zju.edu.cn (Q.G.); shengk@zju.edu.cn (K.S.)

* Correspondence: ren_na@zju.edu.cn; Tel.: +86-571-8795-1345

Received: 08 May 2020; Accepted: 08 June 2020; Published: date

---

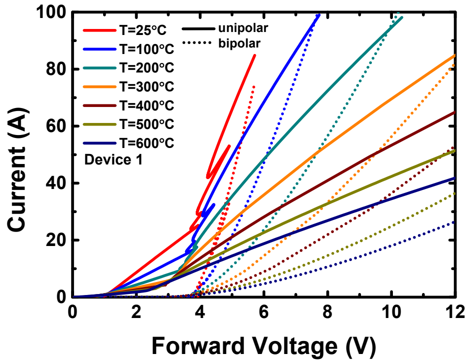

(**a**)

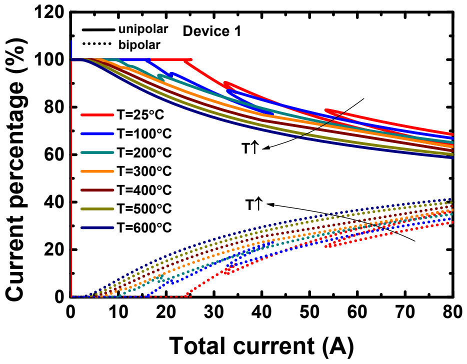

(**b**)

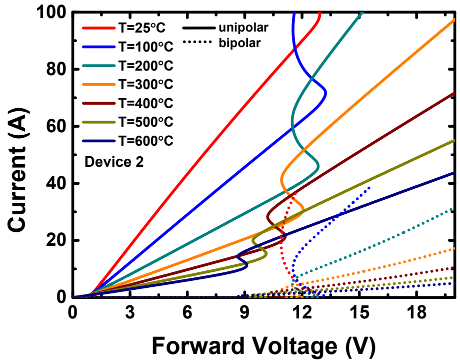

(**c**)

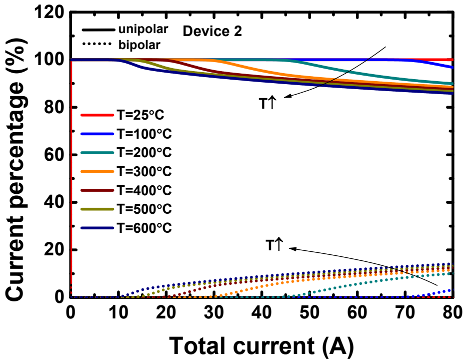

(**d**)

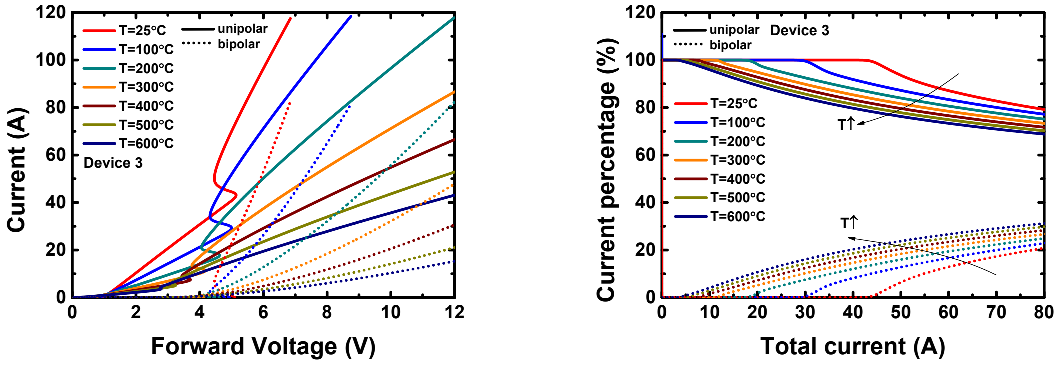

(**e**) (**f**)

Figure S1. The simulated static forward characteristics, with unipolar and bipolar current plotted separately, of (**a**) Device 1, **(c**) Device 2, and **(e)** Device 3 at *T* = 25–600 °C. The percentage of unipolar and bipolar current in total current of (**b**) Device 1, (**d**) Device 2, and (**f**) Device 3, extracted from (**a**), (**c**) and (**e**).

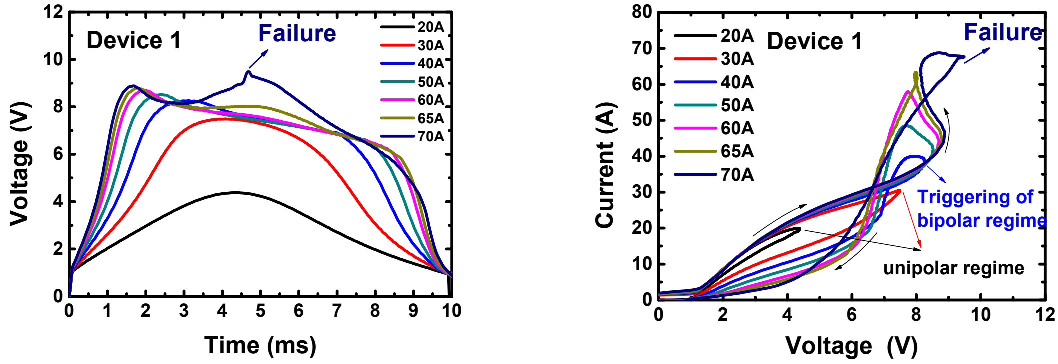

(**a**) (**b**)

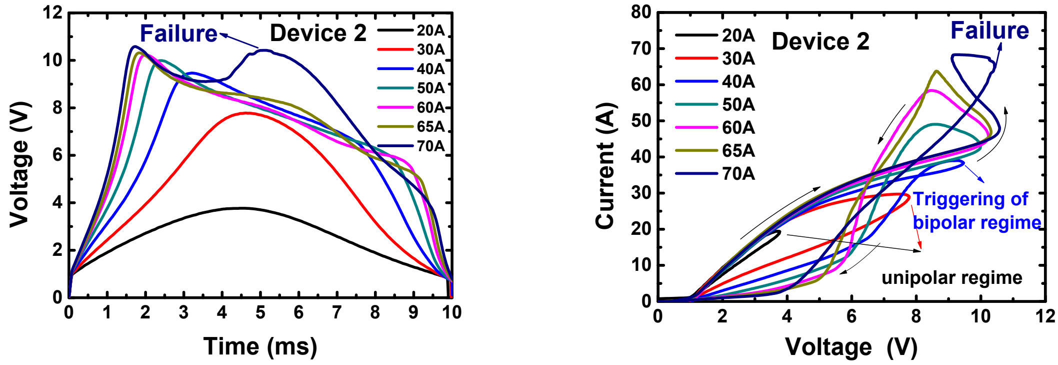

(**c**) (**d**)

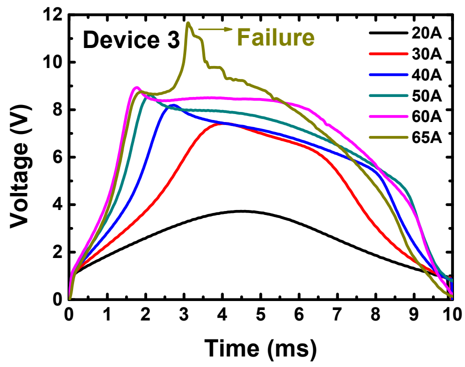

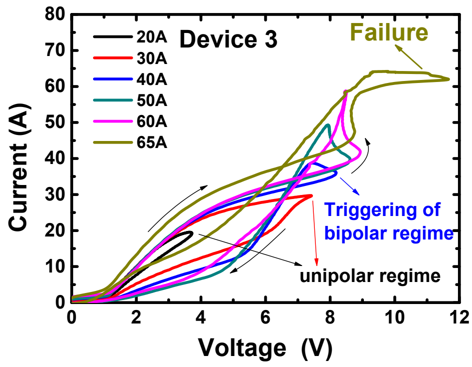

(**e**) (**f**)

Figure S2. Voltage waveforms and I-V trajectories of the three devices during the surge current tests: (**a**)(**b**) for Device 1, (**c**)(**d**) for Device 2, and (**e**)(**f**) for Device 3.

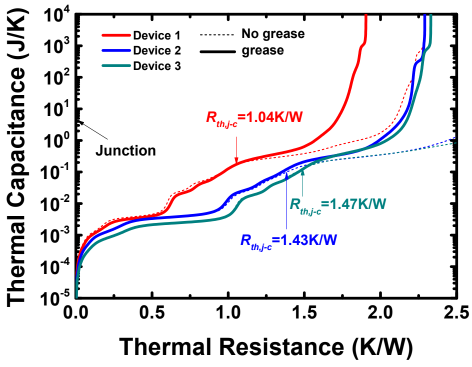

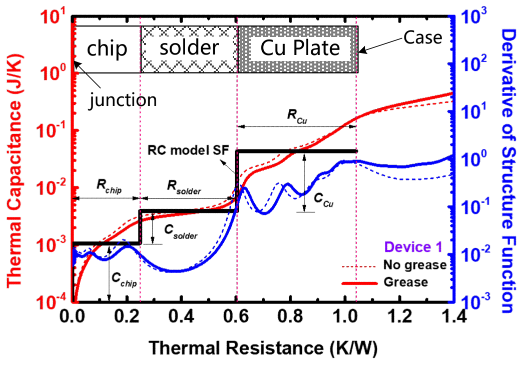

(**a**) (**b**)

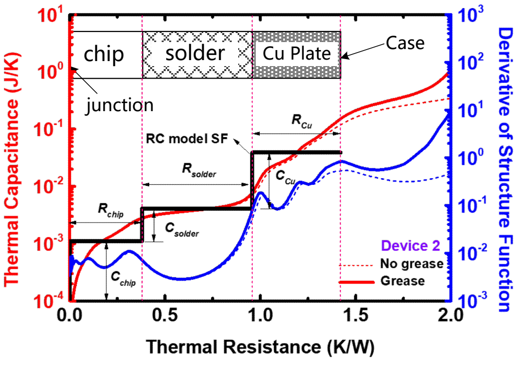

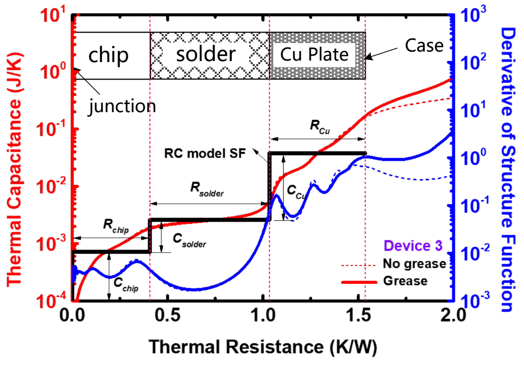

(**c**) (**d**)

Figure S3. (**a**) The structure functions of the three devices. The junction-to-case thermal impedance could be determined by the bifurcation point of the solid and dashed lines. The zoomed-in structure function and its derivative of (**b**) Device 1, (**c**) Device 2, and (**d**) Device 3, as well as the extracted thermal resistance and capacitance of each layer.

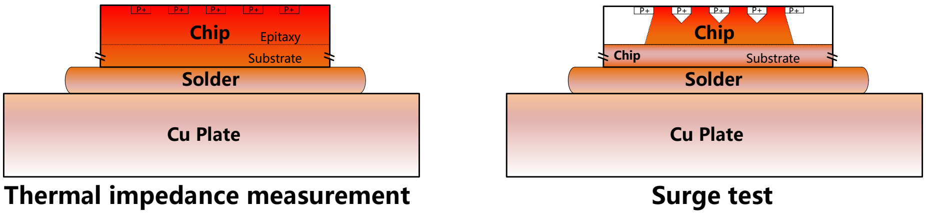

(**a**) (**b**)

Figure S4. The heat paths inside the chip, the solder, and the copper plate of the device during (**a**) the thermal impedance measurement and (**b**) the surge process.
